# Plasma indicators of bovine health: Impacts of diet supplementations and pre-slaughter stress

**DOI:** 10.1016/j.dib.2018.10.009

**Published:** 2018-10-04

**Authors:** Mylène Delosière, Agnès Thomas, Claudia Terlouw, Denis Durand

**Affiliations:** Université Clermont Auvergne, INRA, VetAgro Sup, UMR Herbivores, F-63122 Saint-Genès-Champanelle, France

**Keywords:** Cows, Plasma, Health, Diet, Stress

## Abstract

This data article reports the values of indicators of bovine health determined in the plasma of Normand cull-cows at different times of the about 100 days lasting finishing period and at slaughter. The data constitute a large dataset based on the quantification of metabolites and the evaluation of enzymes activities allowing the determination of antioxidant capacity, oxidative stress level, energy and lipid metabolisms, activity of the Hypothalamo-Pituitary-Adrenal axis and the hepatic status in cull-cows.

**Specifications table**

**Table:** 

Subject area	Biochemistry, Biology
More specific subject area	Animal health, oxidative stress, lipid oxidation
Type of data	Tables, figures
How data was acquired	Spectrophotometry, spectrofluorometry, enzymatique… (precisions in Table 1)
Data format	Analyzed and ready to use
Experimental factors	Indicators were determined on total plasma or protein-depleted plasma
Experimental features	Registration of plasma indicators of bovine health during a finishing period studying diets and pre-slaughter stress treatments on animals
Data source location	INRA, Theix, St-Genès-Champanelle, France
Data accessibility	Dataset is available in public repository: Portail Data INRA (data.inra.fr)
	Data identification number: 10.15454/UUASR4
	https://doi.org/10.15454/UUASR4
	During the reviewing process by Data In Brief, please find data in this private URL:
	https://data.inra.fr/privateurl.xhtml?token=a82e4fab-654b-4d7b-9848-4a2d11bfbe49
Dataset citation	[1] Delosière M., Thomas, A., Terlouw, C., Durand, D., (2018) "Dataset: Plasma indicators of bovine health", https://doi.org/10.15454/UUASR4, Portail Data Inra, DRAFT VERSION
Related research articles	[2] Gobert, M., Bourguet, C., Terlouw, C., Deiss, V., Berdeaux, O., Comte, B., Gruffat, D., Bauchart, D. & Durand, D. (2009). *11th International Symposium on Ruminant Physiology*. Clermont-Ferrand, France.
	https://prodinra.inra.fr/record/32662
	[3] Bourguet, C., Deiss, V., Gobert, M., Durand, D., Boissy, A. & Terlouw, E. M. C. (2010). Characterising the emotional reactivity of cows to understand and predict their stress reactions to the slaughter procedure. *Applied Animal Behaviour Science* 125, 9–21.
	https://doi.org/10.1016/j.applanim.2010.03.008

**Value of the data**

● Data provide 26 plasma indicators of animal health, including oxidative stress evaluation, of 73 finishing Normand cull cows.
● Indicators were obtained throughout the 100 days finishing period and at slaughter.
● Cows were subjected to 3 feeding diets and 2 slaughter protocols relative to stress in an incomplete 3 × 2 factorial design.
● It is the first time a large dataset combines various health indicators in Normand cull cows. This dataset can be used to: assess of values of health indicators in cows; analyze relationships between health indicators to increase our understanding of the metabolism of cows; compared with other data from plasma or tissues of ruminants in order to understand effects of breeds, species, diet supplementation, or preslaughter-stress on health indicators; be computationally aggregated with health indicators of other cows.

## 1 Data

The dataset reports the concentration of metabolites determined in plasma of cows in order to evaluate the health׳s parameters of animals (Table 1) during the finishing period before slaughter. The factors of variation were diet supplementations and pre-slaughter stress. Plasma sampling took place throughout the about 100 days finishing period: the day before experimentation, on days 50 and 100 and just after stunning, before bleeding. Details of the sampling procedures can be found in Bourguet et al. [3] and Gobert [4].

**Table 1:** Plasma indicators of bovine health.

**Indicators**	**Abbreviations (Unit)**	**Biological meanings**	**Methods**	**Technologies**	**Equipments**	**References**
**Indicators of antioxidant capacity**						
Kinetics of conjugated dienes (CD) generation	Lp (min)	Resistance time of PUFA against peroxidation	*ex vivo*	Spectrophotometry	Uvikon XS	[5]
1 - Length of lag phase						
Kinetics CD generation	Rmax (A_234_/min)	Speed of the propagation reaction of PUFA peroxidation	*ex vivo*	Spectrophotometry	Uvikon XS	[5]
2 - Maximal rate of peroxidation						
Kinetics CD generation	CDmax (A_234_)	Quantity of peroxidabilizable PUFA	*ex vivo*	Spectrophotometry	Uvikon XS	[5]
3 - Maximum amount of CD accumulated after the propagation phase						
Total Antioxidant Status	TAS (mmol TEAC/L)	Antioxidant capacity determined comparatively to "trolox equivalent antioxidant capacity" (TEAC)	*ex vivo*	Spectrophotometry	Uvikon XS	[6] adapted by [7]
Vitamin A	Vit A (µg/mL)	Lipophilic antioxidant	*in vivo*	High performance liquid chromatography	HPLC Kontron Sys1 – detector UV/Vis	[8]
Vitamin E	Vit E (µg/mL)	Lipophilic antioxidant	*in vivo*	High performance liquid chromatography – detector UV/Vis	HPLC Kontron Sys1 – detector UV/Vis	[8]
Antioxidant Capacity of water-soluble-portion	ACW (nmol/mL)	Antioxidant capacity of "water-soluble portion" comparatively to ascorbic acid capacity	*in vivo*	Emission spectroscopy	Photochem Analytic jena	[9]
Antioxidant Capacity of lipid-soluble portion	ACL (nmol/mL)	Antioxidant capacity of "lipid-soluble portion" comparatively to Trolox capacity	*in vivo*	Emission spectroscopy	Photochem Analytic jena	[9]
**Indicators of oxidative stress level**						
Malondialdehyde	MDA (µg/mL)	End-product of PUFAs (bearing more than 2 unsaturations) oxidation	*in vivo*	High performance liquid chromatography – fluorescence detector	HPLC Perkin – Serie 200 – Fluorescence detector	[10]
Free 4-Hydroxy-2-nonenal	4-HNE (ng/mL)	End-product of n-6 PUFA oxidation	*in vivo*	Gaz chromatography – mass spectrometry	Watters quattro micro	[11] adapted by [2]
Free 4-Hydroxy-2-hexenal	4-HHE (ng/mL)	End-product of n-3 PUFA oxidation	*in vivo*	Gaz chromatography – mass spectrometry	Watters quattro micro	[11] adapted by [2]
**Indicators of energy and lipid metabolism**						
Triacylglycerols	TG (mg/dL)	Lipid metabolism	*in vivo*	Spectrophotometry	UVK-LAB Biochrom Libra S22	[7]
Phospholipids	PL (mg/dL)	Lipid metabolism	*in vivo*	Spectrophotometry	UVK-LAB Biochrom Libra S22	[7]
Total Cholesterol	TC (mg/dL)	Lipid metabolism	*in vivo*	Spectrophotometry	UVK-LAB Biochrom Libra S22	[7]
Free cholesterol	FC (mg/dL)	Lipid metabolism	*in vivo*	Spectrophotometry	UVK-LAB Biochrom Libra S22	[7]
Cholesteryl esters	CE (mg/dL)	Lipid metabolism	*in vivo*	Spectrophotometry	UVK-LAB Biochrom Libra S22	[7]
Non-esterified fatty acids	NEFA (mg/dL)	Lipid metabolism	*in vivo*	Spectrophotometry	UVK-LAB Biochrom Libra S22	[7]
Apolipoprotein A1	ApoA1 (mg/dL)	Apolipoprotein characteristic of High Density Lipoprotein (HDL)	*in vivo*	Simple radial immunodiffusion technique of Mancini		[12]
Glucose	Glucose (mg/dL)	Energetic metabolism	*in vivo*	Spectrophotometry	UVK-LAB Biochrom Libra S22	[13]
Beta-hydroxybutyrate	β-OH (mmol/L)	Energetic metabolism	*in vivo*	Spectrophotometry	UVK-LAB Biochrom Libra S22	[14]
Lactate	Lactate (mmol/L)	Energetic metabolism	*in vivo*	Spectrophotometry	UVK-LAB Biochrom Libra S22	[15]
**Indicator of the activity of the Hypothalamo-Pituitary-Adrenal axis**						
Cortisol	Cortisol (ng/ml)	Hypothalamic-pituitary-adrenal axis activity	*in vivo*	Radioimmunoassay	Compteur gamma Cobra II Packard	[16]
**Indicators of liver status**						
Alanine aminotransferase	ALAT (IU/L)	Enzyme in relation with hepatic functions	*in vivo*	Spectrophotometry	Uvikon XS	Sobioda kit (RC1160-04)
Asprate aminotransferase	ASAT (IU/L)	Enzyme in relation with hepatic functions	*in vivo*	Spectrophotometry	Uvikon XS	Biodirect TGO kit (RC1157-02)
Phosphatase alkaline	PAL (IU/L)	Enzyme in relation with hepatic functions	*in vivo*	Spectrophotometry	Uvikon XS	Thermo scientific kit (ref: 981771)
Gamma-glutamyl transpeptidase	GGT (IU/L)	Enzyme in relation with hepatic functions	*in vivo*	Spectrophotometry	Uvikon XS	Biodirect kit (RC1124-03)

## 2 Experimental design, materials and methods

### 2.1 Animals

Seventy-five, 48–60 months old, Normand cull cows (mean live weight 642 kg) were introduced into the experiment organized in three repetitions, conducted in winter 2007 (*n* = 24), summer and autumn 2007 (*n* = 25) and winter 2008 (*n* = 26). Cows were identified by numbers between 251 and 331. Two cows presenting health problems during the finishing period (sarcosporidiose and abomasum flipping) were removed from the experimentation leaving 73 cows.

### 2.2 Dietary treatments

**Fig. 1:**
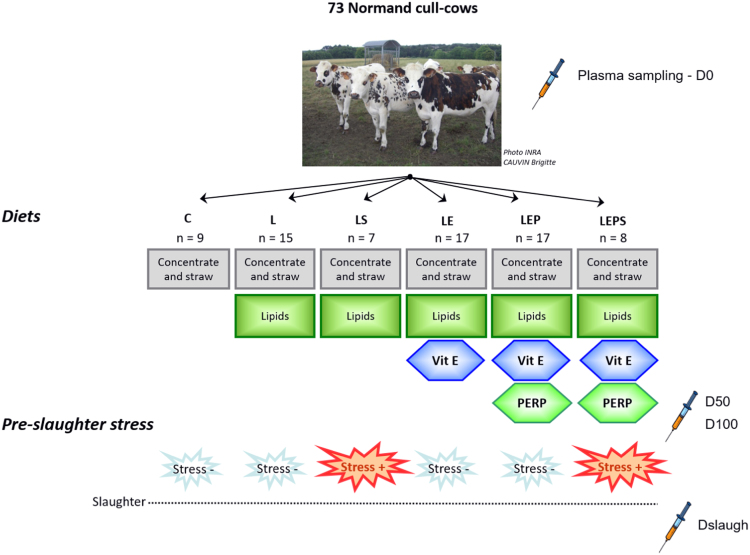
Experimentation designed to study the effects of 3 diet supplementations (lipids, lipids and vitamin E, lipids, vitamin E and PERP) and 2 slaughter protocols relative to pre-slaughter stress conditions on plasma of Normand cull-cows over a 100 days finishing period and at slaughter.

All cows were given a straw (30%) and concentrate (70%)-based diet during 101 ± 3 days (Fig. 1). Nine cows received no supplementation (Control group, **C**). For 64 of the cows the diet was supplemented with lipids (40 g oil/kg diet DM, **L** group) provided by extruded oilseeds (Table 2). For the cows supplemented with lipids, 17 cows received a diet supplemented with vitamin E (155 IU/kg) (**LE** group) and 17 cows with vitamin E (155 UI/kg) and plant extracts rich in polyphenols (PERP; 7 g/kg diet DM) (**LEP** group). The PERP were prepared from rosemary (*Rosemarinus officinalis*), grape (*Vinis vitifera*), citrus (*Citrus paradisi*) and marigold (*Calendula officinalis*) by the Phytosynthèse company (Riom, France) (INRA patent #P170-B-23.495 FR). Cows were housed in 6 × 6 m pens with straw bedding combining 2 experimental and 2 non experimental animals according to a balanced design relative to feeding treatments. Pens were equipped with electronic feeding gates and individually offered the appropriate allowance of concentrates and straw per day for each cow. This periodically adjusted allowance supplied the required amount of nutrient to achieve a target growth rate of 1150 g/d. The finishing period of 101 d. in our study was slightly longer than the 70 d. French standards for cattle and was chosen to facilitate experimental organization and to achieve good production conditions in such cull-cows.

**Table 2:** Experimental composition of the diets. Cows were given a straw-concentrate-based diet (C, *n* = 9), or the same diet enriched with lipids rich in PUFA from extruded linseed and rapeseeds (40 g oil/kg diet DM) provided alone (L, *n* = 22) or with vitamin E (155 IU/kg diet DM) (LE, *n* = 17) or with vitamin E plus plant extracts rich in polyphenols (7 g/kg diet DM) (LEP, *n* = 25).

Item	Experimental diets			
	C	L	LE	LEP
Total Dry Matter Intake (kg/day)	13	13	13	13
Straw (% DMI)	30	30	30	30
Concentrate (% DMI)	70	70	70	70
Ingredients detail (% of DMI)				
Total Lipids	1.9	5.9	5.9	5.9
including PUFA	1.38	4.85	4.85	4.85
Calcium	0.12	0.19	0.19	0.19
Phosphorus	0.24	0.38	0.38	0.38
Vitamin E (IU)	0.9	0.9	15.5	15.5
PERP	0.0	0.0	0.0	0.7

### 2.3 Pre-slaughter treatments

**Fig. 2:**
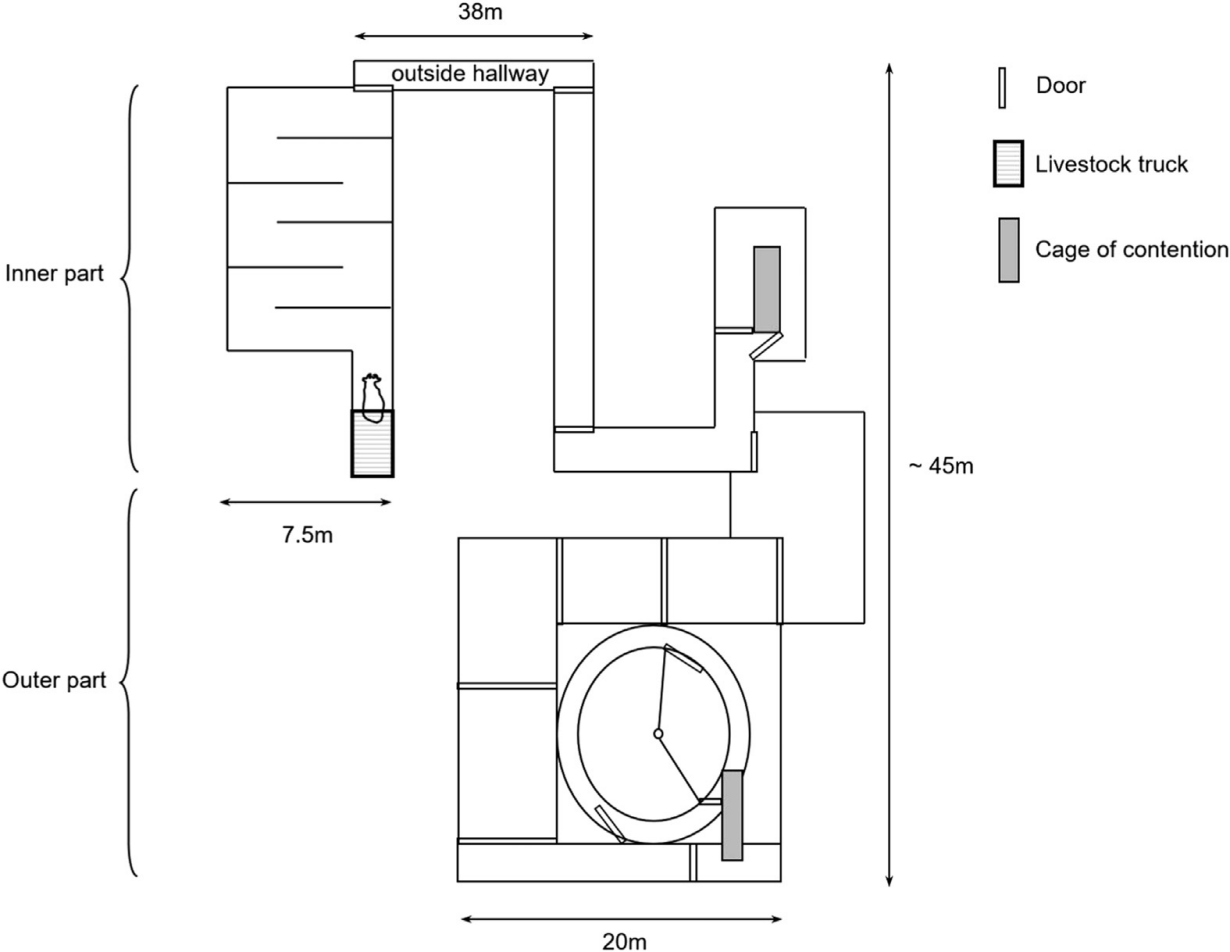
Schematic representation of the labyrinth used for the slaughter protocol with “moderate stress” conditions. The labyrinth was partly indoors and partly outdoors and included corridors with sharp bends and two cattle crushes in which the animal was repeatedly restrained. See [3] for details.

At the end of the finishing period, two slaughter conditions were used: limited stress (stress −) *vs* moderate stress (stress +), which are described in detail in Bourguet et al. [3]. Of each of the L and LEP dietary groups, 7L and 8 LEP cows were slaughtered under the moderate stress conditions, respectively, referred to as the **LS** and **LEPS** cows. All other cows were slaughtered under limited stress conditions. Briefly, each cow slaughtered under limited stress conditions was directly transported (6.9 ± 0.6 min) in a lorry (3 × 2 m) from the experimental farm towards the experimental abattoir of the INRA research center. During the journey of 2 km, each cow was accompanied by a non-experimental conspecific to avoid social isolation stress and were handled calmly. The objective of the moderate stress treatment was to combine in a standardized manner stress factors of psychological (novelty, social isolation, presence of active humans, noise) and physical origins. Each cow was individually transported (11.3 ± 0.3 min) towards an unfamiliar farm where a labyrinth was constructed with communicating corridors containing many sharp bends (Fig. 2). The labyrinth was partly indoors (138 m) and outdoors (122 m). Cows were unloaded at the entrance of the indoor part of the labyrinth and taken through it 3 times, by 2 purposely noisy experimenters (shouting and hitting metal structures with a stick) over a period of 28 min. They were subsequently individually transported (14.4 ± 0.8 min) to the experimental abattoir where they were immediately unloaded. Upon arrival at the abattoir, all cows were immediately stunned by a captive bolt and bled.

Cows were slaughtered with one slaughter day per week over a period of 8 weeks. Slaughter took place between 07:30 and 11:10. Each slaughter day, two experimental cows from a same pen were slaughtered using each slaughter condition.

### 2.4 Sample collection

Blood samples were collected in the morning, before food distribution day 0 (**D0**) and after the beginning of food distribution day 50 (**D50**) (not all cows) and day 100 (**D100**). The collection by venepuncture used tubes containing anticoagulants, such as trisodique ethylene diamine tetraacetique (EDTA K3), lithium heparin or trisodique citrate and were placed on ice until centrifugation. Blood samples were collected immediately after stunning (before bleeding) (**Dslaugh**) by venepuncture syringe and blood was distributed in tubes containing EDTA K3 or lithium heparin and placed on ice until centrifugation. The tubes were centrifuged at 4500 rpm (corresponding to 1950 g) during 15 min (+4 °C), and plasma was transferred to Eppendorf tubes which were stored at −80 °C. Some tubes were acidified using HClO_4_ to achieve a deproteinization needed for β-hydroxybutyrate and lactate determinations. Some plasma tubes presented hemolysis and discarded explaining some missing values in the dataset.

Biochemical approaches were applied on the plasma in order to appreciate the global health of cows evaluating indicators of antioxidant capacity, oxidative stress level, energy and lipid metabolisms, Hypothalamo-Pituitary-Adrenal activity and hepatic status (detailed in Table 1).
