# Supplementary material for: Plasma indicators of bovine health: Impacts of diet supplementations and pre-slaughter stress
Source: Data Brief. 2018 Oct 4;21:2496–503. doi: 10.1016/j.dib.2018.10.009 (PMC6288407; doi:10.1016/j.dib.2018.10.009)
Supplement: Supplementary file 1 — Transparency document [file mmc1.docx]

Competing interests

Authors declared no competing interests.
